# ‘Keeping it real’: A qualitative exploration of preferences of people with lived experience for participation and active involvement in mental health research in Australia

**DOI:** 10.1111/hex.13934

**Published:** 2023-12-18

**Authors:** Julia Dray, Victoria J. Palmer, Michelle Banfield

**Affiliations:** ^1^ Centre for Mental Health Research, National Centre for Epidemiology and Population Health The Australian National University Acton Australian Capital Territory Australia; ^2^ The ALIVE National Centre for Mental Health Research Translation The University of Melbourne, The Australian National University Melbourne Victoria Australia; ^3^ The Department of General Practice and Primary Care, Primary Care Mental Health Research Program Melbourne Medical School, Faculty of Medicine, Dentistry and Health Sciences Melbourne Victoria Australia

**Keywords:** involvement, lived experience, qualitative research methods, research participation

## Abstract

**Background:**

Historically, researchers have been apt at conducting research on, rather than with, the people who are the focus of their efforts. Such approaches often fail to effectively support and benefit the populations they are intended to. This study aimed to explore the preferences of people with lived experience for engagement with research either as research participants within studies, or through active involvement in mental health research.

**Methods:**

Data for this paper were collected in three separate lived experience agenda‐setting studies conducted over a 9‐year period from 2013 to 2022; two group discussions and an open‐ended online survey. Data were combined and thematic analysis undertaken.

**Results:**

Participants described the inclusion of lived experience as a critical ingredient and the highest level of knowledge and expertise in mental health research that should lead to knowledge generation and research agendas. Participants discussed the importance and value of research that enables sharing experiences and stories, expressed a need for flexibility in research methods for choice and agency, and support for greater active involvement of people with lived experience across all stages of research. Participants also spoke to the need for perspective and knowledge generated from people with lived experience to have equal power in research, making space for lived experience voices across multiple aspects of research, and greater respect and recognition of the value of lived experience.

**Conclusion:**

Lived experience in mental health research is coming of age, but dedicated, cocreated development is needed to get it right. People with lived experience increasingly understand the value their experiential knowledge brings to the mental health research effort, and describe a wide range of ways that researchers can support them to be research participants, and to get actively involved. Power‐sharing, respect and recognition of lived experience as central to effective mental health research are the keys to ‘keeping it real’.

**Patient or Public Contribution:**

People with lived experience of mental health problems or distress either personally, and/or as carers, family and kinship group members, were involved in the coideation and codesign of this research. All authors identify as people with lived experience.

## 1 INTRODUCTION

Historically, researchers have been apt at conducting research on, rather than with, the people who are the focus of their efforts. Such top‐down, research‐driven approaches are often to the detriment of participants and their needs, and contribute to disempowerment and marginalisation.^1^, ^2^ Without engagement with people who the research is about, research processes, methods and findings will continue to fail to effectively support and benefit the populations for which they are intended.^3^ In mental health research, the people for which research is intended are people with lived experience of mental health problems or distress, either personally and/or as carers, family and kinship group members. We recognise that a broad range of preferred language exists to describe lived experiences: consistent with the terms used over the course of the research studies reported in this paper, we hereafter respectfully refer to people who identify as having personal lived experience as ‘consumers’, and carer, family and kinship group members as ‘carers’. Further, we use the term ‘engagement’ in research to refer to both participation (providing research data) and involvement (actively contributing to research), and ‘lived experience researchers’ to refer to those who combine their lived experience with formal or active research training to conduct mental health research.

Researchers have recently begun to understand the importance and benefits of engagement with all stakeholders through codesign or coproduction of research. This includes people from the population of interest for research, consumers and carers in mental health contexts, health service staff and policy makers.^4^, ^5^ Examples of advocacy for more widely engaging people with lived experience in service and system design, policy development and research are emerging internationally (e.g., the WHO Framework for meaningful engagement of people living with noncommunicable diseases and mental health and neurological conditions),^6^ and in Australia (e.g., the National Health and Medical Research Council and Consumers Health Forum of Australia Statement on Consumer and Community Involvement in Health and Medical Research and the subsequent development of a Consumer and Community Involvement Toolkit).^7^, ^8^ While this growth in recognition of the importance of lived experience is promising, involvement in design and implementation processes, and in collection and analysis of research findings, remains uncommon.^2^, ^9^

The ‘why’ for embedding lived experience has also been increasingly documented in research in recent years. Inclusion of lived experience as a knowledge source in its own right in research has been found to improve outcomes for people with mental health problems, such as increased connectedness, confidence and hope, a positive sense of belonging and culture, and increased knowledge and skills.^5^, ^10^ This experiential lens and expertise helps ensure research is tailored to the needs and preferences of the people whom the research is about,^11^ improves research design, meaning and impact^5^ and offers an opportunity for multiway capacity building between people with lived experience and researchers.^2^, ^5^ Active involvement also addresses epistemic injustice, where the voices of people who experience mental health issues are frequently silenced, dismissed as not valid, or overridden by other, more powerful professional voices.^12^ However, less is known about ‘how’ people with mental health problems would like to be engaged across research and its related processes, with limited research directly asking people with lived experience of mental health problems about such preferences.^2^ We aimed to qualitatively explore preferences of people with lived experience for engagement in research. Our primary focus was on active involvement in research design, data collection and analysis, writing and dissemination and translation activities; however, participants also shared ideas for improving engagement with intended research participants.

## 2 METHODS

The current paper draws on data from three separate Australian lived experience agenda‐setting studies, conducted across a 9‐year period from 2013 to 2022, held with consumers, carers and people who indicated having both experiences. It brings together findings from Study 1: a large group forum (2013); Study 2: two online World Cafes (2021) and; Study 3: a national priority‐setting survey (2022). The ethical aspects of the research were approved by the Australian National University Human Research Ethics Committee (protocol 2013/388). All participants provided written or online informed consent.

### 2.1 Study 1

In November 2013, ACACIA: The Australian Capital Territory (ACT) Consumer and Carer Mental Health Research Unit held our inaugural event to develop a research agenda and methods for effective partnership with people with lived experience in the ACT. Methods for the whole day are described in detail elsewhere.^2^ The forum was entirely lived experience‐led, organised and run by the ACACIA lived experience research team and Consumer and Carer Advisory Group. Participants were recruited via advertising to the local consumer and carer peaks, and from a register of people with lived experience who had previously expressed an interest in mental health research participation maintained by our research Centre. For the component focused on methods of active involvement in research, we conducted a 1‐h facilitated large‐group discussion. The forum facilitator asked participants to suggest ways of ensuring that people with lived experience of mental health issues were actively involved in the research process, alongside their preferred ways to gain research skills. To help frame the discussion, the research process was illustrated as five key stages, as conceptualised by the National Health and Medical Research Council^7^, ^13^: deciding what to do, deciding how to do it, doing it, letting people know the results and knowing what to do next. Lived experience researchers took notes during the discussion, which comprised the Study 1 data included in analyses.

### 2.2 Study 2

In April 2021, our team conducted two online World Cafes to update the lived experience research agenda and gather further data on preferences for engagement with mental health research.^14^, ^15^ Procedures for the World Cafes, including challenges and adaptations to the method, have been published in detail.^15^ In brief, this method involved bringing two groups of participants together online (our virtual ‘café’) to discuss particular issues, and then rotating participants into new online rooms (our ‘café tables’), with new participants to discuss new issues. Participants were recruited through national mental health consumer and carer networks and social media in Australia. The online groups were facilitated by lived experience researchers, and ran for approximately 2.5 h. For the discussion on methods of engagement, participants were asked ‘How do you currently engage with research?’ Follow up prompts included ‘how would you like to engage with research in the future,’ ‘how would you like to be informed about how to help with being involved in conducting research’, ‘what features of research do you think make it useful for you or for others’ and ‘how do you find out about participating in mental health research?’ The interactive online polling platform Slido (sli.do s.r.o, Bratislava, Slovakia) was used for participants to enter responses to the discussion questions, which formed word clouds that were then used to prompt further discussion. Responses were downloaded as Excel files for inclusion in the analysis.

### 2.3 Study 3

We conducted our third study of preferences for involvement in early 2022, as part of the ALIVE National Centre Priority‐Setting Survey. The online open‐ended survey was conducted using the Qualtrics platform (Qualtrics, Provo, UT), and was advertised through consumer and carer networks, social media and a distribution list of 257 mental health organisations nationally, including lived experience peak organisations (such as Australian nongovernment and community organisations, and advocacy groups representative of people with lived experience of mental health problems) in states and territories. The survey comprised sociodemographic questions, followed by two open‐ended questions: ‘Please share three things that mental health research should focus on’ (priorities for research) and ‘Please share three ways you feel that lived‐expertise from either consumer and/or carer (depending on your own experiences) could be included in mental health research’ (involvement in research). Data from both questions were downloaded as an Excel file, with only data from the second question analysed for the current paper.

### 2.4 Data analysis

All data were imported into NVivo 12 (QSR International) and inductive thematic analysis completed by one member of the research team, with regular meetings and review of the developing structure with a second member of the team.^16^, ^17^, ^18^ Codes were generated using an inductive approach to derive meaning from the data and guide theme generation. An iterative process was undertaken, with initial codes summarised, multiple rounds of additional coding completed, with codes then reviewed and connected for refinement into the final presented themes by discussion with the team. Alongside engagement with research, participants also discussed participation outside research contexts including lived experience of clinical care, service and system design and redesign, clinician knowledge and understanding of lived experience and lived experience of being part of clinical trials. These topics are outside the scope of the current study and are therefore not discussed here but will inform wider activities of the ALIVE National Centre for Mental Health Research Translation and its focus on system redesign, improving experiences of care and implementation of research to address unmet needs.

## 3 RESULTS

### 3.1 Participant characteristics

Participants in Study 1, the group forum, comprised 24 people from the ACT with lived experience as either a consumer (*n* = 14), carer (*n* = 5) or with both perspectives (*n* = 5) who were recruited via advertisements to local consumer and carer networks. For Study 2, 11 people with lived experience participated across the two online groups, and were from Australia's eastern mainland (New South Wales, Victoria, Queensland and ACT). To maintain confidentiality with the small numbers of participants in Studies 1 and 2, no further specific demographic data were collected, but during discussions participants identified as from a range of age groups and gender identities.

Participants in Study 3, the national priority‐setting survey, comprised 365 people with lived experience as consumers (*n* = 207), carers (*n* = 52) or both (*n* = 106). The majority of participants identified as female (*n* = 280, 77%), with 18% (*n* = 65) identifying as male, 4% (*n* = 17) using another term and 1% (*n* = 3) who preferred not to say. Ages ranged from 20 to 93 years (*M* = 46.5, SD = 14.5). The majority of participants were from metropolitan areas (*n* = 242, 66%), and over half were from Victoria (*n* = 134, 37%) and New South Wales (*n* = 79, 22%). However, we did receive responses from all Australian States and Territories and all categories of remoteness (metropolitan through to very remote communities) based on the Modified Monash Model.^19^ Participants were also invited to share how they described their lived experience in their own terms. The wide range of ways in which people chose to respond to this question precluded meaningful tallying of responses. The range included references to diagnoses such as depression, complex posttraumatic stress disorder, borderline personality disorder, bipolar disorder and schizophrenia; other terms such as stress, grief, family violence and suicidality; or descriptors such as ‘complex’, ‘unmet long term [needs]’ and ‘exhausting’. The majority of participants listed more than one condition or descriptor. We now report on the themes that were identified from the analysis of the pooled datasets using theme areas and quotes from participants to illustrate further their meanings and the nuances of what preference for participation and active involvement mean for them.

### 3.2 Preferences for participation and active involvement in mental health research

#### 3.2.1 Lived experience as a critical ingredient

Lived experience was noted as a critical ingredient when conducting research by a large number of participants. Participants described inclusion of lived experience across all research as integral, noted the principle of ‘nothing about us without us’, a need to involve and represent lived experience more broadly across research and policy than is currently the case, and a need for ‘real insights’ helping to set research agendas, steer the research and provide researchers and policy makers with a different and important perspective:

I think that it's invaluable to have lived experience. To let you know how we feel about our lives and what we need and want.

Lived experience know more about the effects of their mental ill health more than any doctor or mental health practitioner, listen more to people with a lived experience.

Lived experience was described as the highest level of knowledge and expertise in mental health research, with a desire to change the perceived clinical research focus:

Life is lived with multiple forces acting on a person. We do not live in a lab or test tube. So lived experience trumps clinical research anytime.

Recognise lived experience as a form of qualified expertise.

Lived experience is as valuable as academic knowledge.

Participants also described knowledge that is lived experience led. For this people noted lived experience as something that should determine the design of research questions and topics and future needs and directions of mental health research:

Lived experience of mental health services and supports, what has worked, what can work and why current systems and supports often fails should inform and be a vital part of the setting of research agendas and research questions.

We have ideas of what we wish was available to us when we've needed it—special insight into the design of the future.

#### 3.2.2 Moving from research participation to active involvement

Across the data sources, many participants expressed support for the engagement of people with lived experience as both research participants and actively involved in processes, such as in coresearcher positions:

Lived experience people both as researchers and participants can greatly help inform research that is more likely to meet the needs of those the research is seeking to develop intervention for.

Highly participatory research methods—[lived experience] experts engaged as coresearchers.

Active involvement was often spoken of as the need to create more formal, identified and paid lived experience roles across all aspect of research and types of organisations involved, including clinical settings, community‐managed organisations and more traditionally research‐heavy institutions such as Universities:

Include lived experience into research settings that typically ignore it—e.g., consumers and carers collaborating with statisticians analysing mental health data, with people designing clinical trials… Lived experience should be represented more broadly beyond its current scope where it gets the most attention in qualitative mental health & health services research.

Universities to ensure all health/mental health faculties have designated lived experience researcher positions…

Participants also spoke of the value and importance of involving carers and families as both research participants, and as actively involved in: research design and conduct, including formulation of research topics and agendas; and addressing unmet service, support and research needs of both consumers and carers:

Having first hand accounts from people with mental health conditions and their carers is a good way of assessing care needs against the current failing health system.

Carer research could be more integrative so it is not perceived merely as a type of ‘service’ to the person.

Processes such as codesign and coproduction were frequently referred to as preferred ways to involve people with lived experience, with agreement that lived experience should be embedded across all aspects and stages of research, with opportunities for leadership:

Leadership across all aspects of mental health research.

Research created by and with those with lived experience.

Processes of analysis, write up and dissemination were a particular stage of research spoken about in detail by participants. This included: discussion of people with lived experience having coauthorship or reviewing and commenting on preliminary findings; making results more accessible including through increased use of plain language, shorter formats, creative mediums, feedback from people with lived experience, open access publication and sharing directly back to participants; consumer and carer translation and/or analysis of collected data; and through use of other dissemination methods such as including people with lived experience as guest presenters at public gatherings and large community or education events:

Participants are included in the reviewing stage of research to ensure the lived experience voice is heard accurately in the research paper's end result.

Get people with lived experience to help with research translation so that any plain language materials are accessible.

‘Keeping it real’ and not excessively theoretical.

#### 3.2.3 One size doesn't fit all: Flexibility, choice and agency

Reflecting the desire for respectful and meaningful engagement, there was huge variation in personal preference for methods that may encourage greater engagement of mental health consumers and carers in research. This emphasised the importance of building flexibility into research design, ensuring choice and agency remain with people with lived experience:

There is no one size fit all in mental health.

Give options for levels of participation—sometimes people will be happy to do an interview, or participate in a group discussion, other times they might just be happy to drop by and chat with someone, or make a phone call etc.

Suggested research methods that may encourage engagement as research participants included: telephone, online or face‐to‐face forums and interviews, both individually and in groups; coreflection groups with unstructured interviews; focus groups and facilitated face‐to‐face discussions; family and carer, input, consultation and participation; home visit fieldwork; written hard copy or online cross‐sectional surveys; longitudinal surveys; crowdsourcing; peer‐led and art‐based research groups; video, audio or other creative arts submissions; case studies or observational studies; narrative approaches; mood diaries or physical health data collected via regular app‐based methods; mixed methods quantitative and qualitative data collection; only qualitative or only quantitative data collection; and options for anonymous contributions.

While genuine inclusion across all stages of research was a common thread, participants also suggested a wide range of preferred ways that researchers could improve active involvement in research processes and dissemination. These suggestions included: information sessions; community forums or public lectures; consultation; active, reciprocal community engagement and increased lived experience engagement in developing community resources; open‐source data or papers including an option for community input and participation; online live discussion forums; expert discussion panels; and consumer carer panels.

Participants also noted various barriers to both research participation and involvement such as cost, difficulties with organising public or other transport, knowing where to find information about being a participant or getting actively involved, understanding provided information, and difficulties gathering participants together when using group formats. Suggested ways of addressing these barriers included: use of consumer and carer organisations, peer workers and other health professionals such as pharmacists, psychologists and general practitioners to inform consumers of research opportunities, and to deliver or conduct research with consumers; using online methods and platforms such as surveys, websites, media and social media to make recruitment more engaging and easy to understand; using existing resources in the community such as newsletters, local papers, schools and notice boards; creating a registry of people interested in being involved in applied lived experience research; having people with lived experience supporting participants; and accessible formal counselling services (e.g., low or no cost, wide hours of availability, options for remote support):

Make it easier for consumers and carers to participate in trials (i.e., locations of trials, ease of access, financial costs involved in participation).

Utilise peer support workers in placement for information collection.

Many participants particularly described peer workers as invaluable for support of research design, implementation and dissemination:

Train lived experience workforce to identify and develop action research coauthored by participants.

Consulting peers at all stages of the research.

#### 3.2.4 Sharing experiences, stories and backgrounds

The theme of shared experiences, stories and backgrounds was also prominent. Participants discussed the importance and value of research participation that involves sharing experiences and stories. This was described in relation to many aspects of applied lived experience research including:

Sharing experiences of what has worked and not worked through focus groups/written feedback.

Ownership over our own narratives/stories—e.g., if studies find people with schizophrenia are 3x more likely to attempt suicide, include in research a section where people with schizophrenia are interviewed so they can share their own views of why that may be the case, in addition to research/analysis.

Instead of asking standard questions just let people talk and share their experiences.

People with lived experience also described participation in research through sharing experiences and stories together, consumer‐to‐consumer and likewise the value of lived experience (or peer) researchers. Participants referred to common humanity, support and advocacy and increased connectedness and feeling validated when participating in research through sharing stories and experiences with other people with lived experience:

Consumers talk to each other differently than with researchers: consumer to consumer = richer data.

People relate to those who've experienced the same. Not people who have never had mental health issues.

For active involvement in research processes, this extended to the need for a community of practice, where skills‐building amongst people with lived experience could be shared:

Would like more LE [lived experience] community of practice—e.g., discuss research and learn skills together.

Many participants also requested consideration of the personal and individual nature of lived experience, as well as respect for diversity and inclusion in research. This could apply both to research participants and to efforts to actively involve people in research processes:

Engaging with populations within the community for context/framing ‐ e.g. more engagement with CALD [culturally and linguistically diverse] people with mental illness and an understanding of how proposed research impacts them, etc.—some research is too generalised and makes assumptions that may not apply to everyone with mental illness based on their background/experience.

Culturally tailored/adaptive methods for engagement and recruitment of research participants.

Broader engagement with lived experience community (diversity of representation), not just the ‘easy to access’ lived experience experts or most prominent.

#### 3.2.5 Power, voice, value

Participants frequently referred to the concepts of power, voice and value. This was particularly so when active involvement processes were, described as a preference. These processes were seen as beneficial for equal sharing of power across varying aspects such as ethics committees, research team roles, research topic and design, working groups, codesign procedures and remuneration:

People with considerable lived experience should be on all ethics committees and have equal power in these committees…

Consumers as a part of research team with actual power over research methods/how questions are asked/experience of participants in study.

Equity & Justice: Equity of power and pay should be considered from the outset: Codesigning with consumers (even imperfect codesign or participatory engagement efforts) should build capability. Equity of pay must be considered when engaging with consumers (It's not ‘co’ if professionals are being paid and we're not)… Additional compensation relating to the emotional labor involved in codesign and lived experience designated roles should also be considered.

Having people with lived experience sharing power was seen as a way to disrupt established systems and challenge the way research is conducted:

Consumer and carer engagement in mental research can and should inform, challenge and disrupt how academics and funders think about critically think about the mental health research is constructed and delivered.

Challenge the dominant models.

This was also expressed as making space for a lived experience voice across multiple aspects of research (e.g., conception, design, policy, commissioning processes), and promoting respect, recognition and value of lived experience:

Greater appreciation of lived experience on every level, sometimes feels tokenistic.

Lived Experience should be elevated and respected and a part of the decision making and prioritizing of the evidence base.

## 4 DISCUSSION

This study has provided insights into the preferences of people with lived experience for participation and active involvement in mental health research. Qualitative analysis of data combined from three lived experience agenda‐setting studies identified the following five themes: lived experience as a critical ingredient; moving from research participation to active involvement; one size doesn't fit all: flexibility, choice and agency; sharing experiences, stories and backgrounds; and power, value and voice. These themes are unsurprising in the context of current research trends and activities toward greater involvement of people with lived experience in research processes; however, what is surprising is the limited literature base that documents preferences directly from people with lived experience of mental ill‐health, distress and or with experiences as carer, family or kinship group members.

The principle that lived experience is a critical ingredient for keeping research real, and should be viewed as essential expertise to lead knowledge generation is central to embedding lived experience in mental health research.^2^, ^9^, ^14^, ^15^, ^20^ Meaningful and authentic lived experience inclusion framed the other themes, it flowed as a consistent narrative throughout discussion of methods that may improve research participation and active involvement, how to move between participation and involvement, and the importance of shared experiences. Consistent with the other elements of the studies, which were focused on developing priorities for the mental health research agenda,^9^, ^14^, ^15^ participants expressed meaning and authenticity as ‘no one size fits all’. They identified a variety of nuanced and often very specific ways of ensuring that people with lived experience feel safe and supported in sharing their experiences. Many methods of decision‐making in research focus on consensus, forcing people to narrow their choices and compromise; however, people with lived experience consistently emphasise flexibility and choice, and matching topic with method, as demonstrating the centrality of lived experience.^2^, ^15^ In particular, more ‘personal’ methods of data collection such as face‐to‐face focus groups, consumers interviewing other consumers, or seeking researchers from similar cultural or experiential backgrounds are viewed as supportive methods for relating stories and experiences in research.^2^ This charges researchers with embracing what may need to be complex research designs, allowing for a range of data collection methods, and researchers from a variety of backgrounds. However, this increases the likelihood that people with lived experience will want to participate in the research, and may come forward to be actively involved in the processes, resulting in better quality research that not only meets people's needs, but affects change and improves outcomes for people within communities.

The other central principle that is consistently expressed across lived experience research is the need for equal power, making space for lived experience voices across multiple aspects of research to demonstrate respect and recognition of the value of lived experience beyond being research participants.^5^, ^10^, ^21^, ^22^ In a recent scoping review of partnerships between consumers and researchers for evaluation in research, Bird et al.^10^ describe the importance of shifting from participation to contributing members of research teams, including genuine involvement across different research roles and activities. Aligning with the current findings, they noted that power imbalances and a lack of value for lived experience voices are key barriers to effective involvement. Equal power and space for voice, challenging existing power structures, trust and respect, transparent processes, collaborative decision‐making and active research roles such as inclusion on the research team, advisory group or steering committee, are described as fundamental to meaningful involvement in research.^5^, ^10^, ^21^, ^22^ In the current studies, participants further described this as challenging the status quo or dominant models through lived experience involvement, consistent with the principles of lived experience involvement in the delivery of services, development and leadership.^20^

To make the shift to genuinely collaborative research with lived experience at its centre, we need to expand our efforts to support its development. The literature reflects growing initiatives to develop embedded lived experience research models, but participants still called for capacity‐building and training for consumer‐ and carer‐led research involvement,^2^ consumers and researchers working in partnership across all stages of research,^9^, ^11^ the inclusion of more identified lived experience academic roles in Australian and international universities,^3^ and service user leadership in research.^11^ Consistent with a recent framework for understanding lived experience identities,^23^ people believe that the dual identity of people with lived experience as participant and professional needs to be recognised, explored and elevated to demonstrate value and respect. Lived‐experience researchers can sit in a liminal space, able to inhabit both consumer and professional research identities, both a critical ingredient and a dynamic often creating ambiguity and related difficulties.^23^, ^24^ These issues go to the heart of how experiential knowledge is valued within the hierarchies of academia and the dominant research agendas.^25^ As with any venture, related literature has documented other barriers and challenges to the involvement of people with lived experience in research. These include: stigma and lack of value or organisational support,^25^, ^26^, ^27^, ^28^ negative attitudes including unconscious bias towards expertise of, collaboration with, and inclusion of people with, lived experience of mental health problems within workforces^25^; lack of acceptance of lived experience researchers both at consumer and organisational or service levels^24^; tokenistic involvement of people with lived experience to satisfy emerging guidelines and policies^25^; lack of recognition of emotional labour experienced by people with lived experience when contributing to research and service evaluation^25^; and the above previously noted lack of value of lived experience voices^10^ and power imbalances.^10^, ^27^

The current study should be viewed in light of some strengths and limitations. In directly asking people with lived experience of mental health problems their preferences for engagement in research, this study addresses a noted methodological limitation in related literature to date. Although there is some move towards evaluating people's involvement after research projects, asking people about their preferences for involvement directly is not common. As outlined, the current paper draws on data from three separate Australian lived experience agenda‐setting studies in 2013, 2021 and 2022. While bringing together a collection of data through three different studies in many ways can be seen as a strength of the current methodology, it is possible this may also have reduced contextual validity. Data were reviewed for consistency in findings, following which the authors combined all three datasets for thematic analysis. Though older data, the content of the 2013 data set aligns well with that of the more recent 2021 and 2022 datasets, providing comprehensive and contemporary views from participants and supporting the reconciliation of all three datasets into one set of findings for this manuscript. Across all three studies, the research team were academic researchers with lived experience of mental health problems or distress either personally, and/or as carers, family and kinship group members, and it is possible some desirability bias may have been present. However, our methodology did not dictate what involvement should mean to people with lived experience of mental health problems, but rather let perspectives of this be led by participants. The multiple and intersecting identities and experiences of participants are acknowledged throughout and reflect that our study sample included people with a wide range of lived experience and mental health diagnosis. Lastly, due to the scope of the present study, we did not investigate differences in findings for carers and consumers and, therefore are unable to comment on potential differences in preferences for involvement.

## 5 CONCLUSION

Lived experience in mental health research is coming of age, but dedicated, cocreated development is needed to ensure that it is well‐grounded in the perspectives of people with lived experience rather than traditional or theoretical framings, so that we ‘keep it real’. People with lived experience increasingly understand the value their experiential knowledge brings to the mental health research effort. They expect that mental health researchers will also recognise this value and invest in opportunities to elevate that voice to ensure the findings from research are implemented and translated into meaningful outcomes. As noted throughout the discussion, in many instances the current findings align with what has been already repeatedly published in this space to date. This emphasises a need to actively apply the preferences of people with lived experience with rigour, respect and accountability in research^29^ and move from intent to action.^6^ Rather than just hear, it is time to listen, and time to get it right.

In Australia, in our work in The ALIVE National Centre for Mental Health Research Translation, we have embedded lived experience at all levels of governance, research design and activities for reporting and dissemination. More recently, the ALIVE National Centre and its associated Lived Experience Research Collective announced the development of a National Strategy for Lived Experience in Mental Health Research^30^ to progress this need. The strategy will explore issues of identity and roles to develop a typology of lived experience in mental health research, and use codesign with various lived experience and research stakeholders to create the necessary structure to guide the sector on active involvement. We know why we should involve people in our research, so it is now time to support the how.

## AUTHOR CONTRIBUTIONS

**Julia Dray**: Writing—original paper conceptualisation, led original manuscript draft, review and editing; conducted study analysis. **Victoria J. Palmer**: Co‐conceived and designed research studies; Writing—manuscript review and editing; project administration; managed data collection; supervision; funding acquisition. **Michelle Banfield**: Co‐conceived and designed research studies; Writing—original paper conceptualisation, original methods draft, contributed to study analysis, manuscript review and editing; project administration; managed data collection; supervision; funding acquisition. All authors reviewed and approved the final manuscript.

## CONFLICT OF INTEREST STATEMENT

The authors declare no conflict of interest.

## ETHICS STATEMENT

The ethical aspects of the research were approved by the Australian National University Human Research Ethics Committee (protocol 2013/388).

## Data Availability

The data are not publicly available due to privacy or ethical restrictions.
